# Lysenkoism Against Genetics: The Meeting of the Lenin All-Union Academy of Agricultural Sciences of August 1948, Its Background, Causes, and Aftermath

**DOI:** 10.1534/genetics.118.301413

**Published:** 2019-04-01

**Authors:** Svetlana A. Borinskaya, Andrei I. Ermolaev, Eduard I. Kolchinsky

**Affiliations:** * N. I. Vavilov Institute of General Genetics, Russian Academy of Sciences, Moscow 119991, Russia; † St. Petersburg Branch of the S.I. Vaviolv Institute for the History of Science and Technology, Russian Academy of Sciences, St. Petersburg 199034, Russia

**Keywords:** genetics in the Soviet Union, history of science

## Abstract

Progress in genetics and evolutionary biology in the young Union of Soviet Socialist Republics (USSR) was hindered in the 1930s by the agronomist Trofim Lysenko, who believed that acquired traits are inherited, claimed that heredity can be changed by “educating” plants, and denied the existence of genes. Lysenko was supported by Communist Party elites. Lysenko termed his set of ideas and agricultural techniques “Michurinism,” after the name of the plant breeder Ivan Michurin, but they are currently known as Lysenkoism. Although Michurinism opposed biological science, Lysenko took up one academic position after another. In 1929, Nikolai Vavilov founded the Lenin All-Union Academy of Agricultural Sciences and became its head; it directed the development of sciences underpinning plant and animal breeding in the Soviet Union. Vavilov was dismissed in 1935 and later died in prison, while Lysenko occupied his position. The triumph of Lysenkoism became complete and genetics was fully defeated in August 1948 at a session of the academy headed by Lysenko. The session was personally directed by Joseph Stalin and marked the USSR’s commitment to developing a national science, separated from the global scientific community. As a result, substantial losses occurred in Soviet agriculture, genetics, evolutionary theory, and molecular biology, and the transmission of scientific values and traditions between generations was interrupted. This article reviews the ideological, political, economic, social, cultural, personal, moral, and ethical factors that influenced the August 1948 session, and its immediate and later consequences. We also outline current attempts to revise the role of the August session and whitewash Lysenko.

## Abstract (precis)

This article reviews the ideological, political, economic, social, cultural, personal, moral, and ethical factors that determined the conduct of the Lenin All-Union Academy of Agricultural Sciences session in August 1948, as well as the immediate...

SEVENTY years ago, a session of the Lenin All-Union Academy of Agricultural Sciences (VASKhNIL, Vsesoiuznaia Akademiia Sel’skoKhoziaistvenenykh Nauk imeni Lenina) was held in Moscow, from July 31 to August 7, 1948, organized by the Academy’s president, Trofim Lysenko, and approved by Joseph Stalin. State authorities directly interfered with the scientific arguments of biologists and prohibited research, in this case in genetics, a rapidly developing scientific discipline at that time.

The atmosphere at the VASKhNIL session was in telling contrast to that at the Eighth International Congress of Genetics, which took place shortly beforehand (July 7–14, 1948). Scientists from various countries met in Stockholm for the first time after the war to evaluate the state of genetics: “*it marked the commencement* [meaning resumption] *of normal scientific exchanges*” (Bengtsson and Tunlid 2010). In his presidential address, Hermann Muller listed the achievements and outlined the main avenues of further development in genetics. He mentioned fighting against Lysenkoism as one of the main tasks for the global scientific community. He was not mistaken. At the VASKhNIL session 1 month later, genetics was proclaimed an idealistic pseudobiology and antinational science, of no importance to agriculture. The son of an Ukrainian peasant, the agronomist Trofim Lysenko, who was the main political actor at the session, first became important between the 1920s and 1930s.

In the 1920s, Russian genetics made major progress. Its leaders, Nikolai Vavilov, Nikolai Kol’tsov, Yurii Philipchenko, Sergei Chetverikov, Aleksandr Serebrovskii, and Mikhail Zavadovskii, paid attention to pressing problems in the applied domain: the diversity of cultivated plants and their wild relatives, gene geography and variation of farm animals, regularities of gene distribution in different geographic regions, suggesting local adaptation, and science-based breeding. Vavilov believed that the mobilization of global plant resources provided a means to eradicate hunger. He traveled to ∼50 countries across five continents, built a unique collection of seeds to be used in breeding, and organized a broad network of breeding stations throughout the country and a state cultivar testing system (Reznik 2017).

In the 1930s, the State, under Stalin, tightened its grip on science. Demonstrating loyalty to communist ideology became mandatory in any presentation, and requirements were imposed to replace the old intellectual class with new specialists from worker and peasant families. Lysenko’s career was fast-moving, although his ideas lacked a scientific basis, he failed to follow accepted scientific methodology, and, in particular, he denied statistics. However, he proposed allegedly new agricultural techniques to improve yields and reported achievements (which were often imaginary because his techniques were immature or their testing with standard scientific procedures did not confirm their claimed efficacy). To cover his failures, Lysenko repeatedly made new promises of increased yields. In 1935, he presented a mixture of his ideas and proposed agricultural techniques as a new research avenue developing the ideas of the famous Russian plant breeder Ivan Michurin. Michurin designed methods to breed fruit and berry plants via distant hybridization, and his main ideas had nothing in common with Lysenko’s ideas (Goncharov and Savel’ev 2016). Because Michurin had died shortly before that time, Lysenko shamelessly used his name, terming his version of biology Michurinism. Michurinism, combined with the political campaign conducted by Lysenko under the Soviet authorities, is now known as Lysenkoism.

Lysenko was supported by Communist Party elites including Stalin himself. Geneticists had to pit scientific arguments against the political accusations that Lysenko and his adherents published in central newspapers, and pronounced at various meetings and conferences attended by Communist authorities.

Most geneticists and agronomists opposed Lysenko, and many were shot during the nation-wide Great Purge from 1936 to 1938, although the exact numbers of losses in these two groups are unavailable. Nikolai Vavilov was arrested in 1940 and died of starvation in prison in early 1943 (Rokitianskii *et al.* 1999; Pringle 2009; Reznik 2017). However, genetics and evolutionary biology continued to be taught at secondary schools and higher education institutions, research in the fields continued, and biologists continued to criticize Lysenko’s ideas until 1948. The August 1948 session of the VASKhNIL ended all of these. Soviet scientists were told to follow the Lysenko’s concept. The event marked a separation between Soviet biology and global science.

It was not until after Stalin died, and Nikita Khrushchev (who also supported Lysenko) was removed from power in 1964, that genetics returned to educational programs and geneticists were once more able to perform their research in the Soviet Union. However, a tacit ban on criticism of Lysenko persisted until the mid-1980s, and those who tried to investigate the causes and consequences of the August session were prosecuted or driven out of the country (Medvedev 1969; Soyfer 1994). A current list of publications on the August session and Lysenko includes several hundred works (deJong-Lambert and Krementsov 2017) demonstrating how absurd the ideas approved at the session were, and how detrimental the introduction of Lysenko’s agrobiological practice was. The conduct of the August session was associated with Stalin’s attempts to subordinate science to ideology, and establish control over science in the Soviet Union and Eastern Bloc countries. It was also associated with the Cold War and the hope of bringing an end to food shortages by miracle-working agricultural methods, as Lysenko promised, as well as with conflicts among different teams and scientific schools in Soviet biology (Soyfer 1994; Efroimson 1989; Krementsov 1997; Roll-Hansen 2005; deJong-Lambert and Krementsov 2017). It would seem that the “Lysenko affair” has already been scrutinized thoroughly. However, attempts have recently been made in Russia to present the events of August 1948 as a purely scientific discussion, and Lysenko as a genius and the pride of Russian science. This makes it important to recall the events of 70 years ago.

The article briefly reviews the ideological, political, economic, social, cultural, personal, moral, and ethical factors that predetermined the organization of the August session and formed the context in which opposing positions were formed between geneticists, who aligned themselves with contemporary global science, and Lysenkoists, who advocated indigenous concepts, ideas, and methods. Here, we consider in detail the organizers’ motives and goals for the August session, how its main attendees behaved, its consequences for biology in the Soviet Union, and its global significance, ending with a brief account of current attempts to whitewash Lysenko and his ideas of heredity.

## Background and Secret Preparation of the 1948 VASKhNIL Session

In the 1920s, the achievements of Russian genetics were known and recognized abroad. Genetic methods, concepts, and research programs were a matter of intense international exchange. Calvin Bridges, Hermann Muller, and Doncho Kostov worked in the USSR (Union of Soviet Socialist Republics), while Soviet researchers worked in the US (Theodosius Dobzhansky, Izrail Agol, Solomon Levit, Georgii Karpechenko, and Mikhail Navashin) and Germany (Nikolai Timofeeff-Ressovsky and Nikolai Slepkov). The government encouraged scientific contacts to facilitate the emergence of the USSR from isolation after the revolution of 1917. The authorities provided special support to geneticists and breeders, expecting their help in agriculture. William Bateson stated that, after visiting Russia at Nikolai Vavilov’s invitation for the 200th anniversary of the Academy of Sciences, “*the revolutionary government is perfectly sincere in its determination to promote and foster science on a very large scale. Signs were not wanting that science*, *especially perhaps in its applications*, *is regarded by the present governors of Russia as the best of all propaganda*” (Bateson 1925). By its 200th anniversary, the Academy of Sciences included 47 academicians and 10 of them were biologists: botanists, zoologists, physiologists, and one microbiologist. Half of the academicians had joined the academy before the revolution. Nikolai Vavilov joined the USSR Academy of Sciences in 1929. In the same year, he was requested to organize the VASKhNIL session and was appointed as its president. He simultaneously headed the All-Union Institute of the Plant Industry (VIR, Vsesoyuznyj Institut Rastenievodstva) and the Institute of Genetics, the first genetics institute formed by the Academy of Sciences. Vavilov was a member of the USSR Central Executive Committee, which was formally the highest body of Soviet state authority. On February 2, 1930, Vavilov wrote to Dobzhansky, who apprenticed with Thomas Morgan, that “*the conditions of research are continuously improving… The need for geneticists is great… We will foster science. The demand for it is incredible*” (Vavilov 1930).

The First All-Union Congress of Geneticists, Crop Breeders, Seed Producers and Animal Breeders (Leningrad, January 1929) was attended by > 2000 participants. The Soviet government and Communist Party regarded this as an important political event. The country’s leaders sent out welcoming telegrams. Articles about the congress appeared in mainstream newspapers with catchy headlines such as “*Soviet science is on the move to help in the fields*.” However, in the late 1920s, the expedited industrialization and forced collectivization started by Stalin led to a crisis in agriculture, and to hunger. Developments that promised quick results were demanded from scientists (Elina 2017). One such development was a project run by Lysenko, a young agronomist from a Ukrainian peasant family. He studied the effects of lower temperatures on vegetative development in cotton, wheat, rye, oat, and barley at a VIR breeding station in Azerbaijan, supervised by the station head, Nikolai Derevitskii, a prominent specialist in agronomic statistics. Derevitskii encouraged him to use a method designed by Vavilov’s colleague, Gavriil Zaitsev.

Lysenko soon proposed an agricultural technique that he termed *yarovization* (vernalization). He claimed that yields would greatly increase if seeds of winter crop varieties that died in harsh frosts were exposed to lower temperatures before sowing, and then sown in spring in the same way as spring varieties. Vernalization shortened the plant’s vegetative period sometimes resulting in ripening in cold climates, suggesting a potential for increased yields. Lysenko believed that heritable changes arise in plants as a result of vernalization, while geneticists already knew the idea to be false. The Ukrainian People’s Commissar (Minister) for Agriculture, Aleksandr Shlikhter, was interested in Lysenko’s proposition and started promulgating vernalization as a miracle-working technique (Joos 2017). Vernalization had already been studied in the 19th century in the United States (by John Klippart) and Russia (by Efim Grachev), and in the early 20th century in Germany (by Gustav Gassner), but had not been observed to increase yields. Given the demands of the authorities, expert breeders Petr Lisitsyn and Nikolai Tulaikov, the geneticist and breeder Andrei Sapegin, and the plant physiologist Nikolai Maksimov agreed that vernalization was a promising technique and advised comprehensive testing before its wide introduction into agriculture in *Sel’skokhoziaistvennaya gazeta* (Agricultural Paper, November 11, 1929, p. 3).

The authorities ignored their warnings. Immediate implementation was ordered in 1931 and the area of fields planted with vernalized seeds increased rapidly. While geneticists promised to produce new varieties within 4–5 years, Lysenko claimed he could do this in 2–3 years and soon reported his imaginary achievements. Lysenko’s promises were enthusiastically embraced by the authorities because agriculture had been damaged by collectivization, seizures of grain expropriated by authorities, and periodic droughts and crop failures, leading to mass deaths from hunger (Tauger 2001; Davies and Wheatcroft 2004; Kondrashin 2008).

After the crop failures and famine of 1932–1933, when ∼6 million people died, the USSR People’s Commissar for Agriculture, Iakov Iakovlev, requested that Vavilov “*provide full assistance to Lysenko’s work and caters to his needs*” (Esakov and Levina 1987, p. 165), which Vavilov did in good faith. In 1934, Lysenko was elected to the Academy of Sciences of Ukraine and became the director of the Odessa Breeding Genetic Institute. In February 1932, Isai Prezent, head of the Society of Marxist Biologists (Anonymous 1932), joined Lysenko. Prezent became the main ideologist behind Michurinist agrobiology, which rejected the existence of genes and postulated that inheritance can be transformed via “retraining” of plants (Lysenko and Prezent 1935).

By 1935, vernalization proved to be unrealistically laborious, or even harmful, because it decreased seeds’ germination. Lysenko attributed his failures to the work of enemies. The explanation was appealing to Stalin, who had already begun his policies of terror in the USSR. Vavilov was dismissed from the post of president of the VASKhNIL in 1935, with Lysenko taking the post in 1938. While the Great Purge was at its peak, Lysenko openly accused geneticists of hampering his methods and named N.I. Vavilov and G.D. Karpechenko, who both perished later (Lysenko 1936). A meeting of the VASKhNIL Presidium was held in April 1937, and Lysenko complained that VASKhNIL leaders supported his work poorly and that he was forced to ask for help (Nurinov 1937). The VASKhNIL president, A.I. Muralov, and his deputies, A.S. Bondarenko and G.K. Meister, were shot soon afterward. Of the 52 VASKhNIL academicians, 12 were shot on false charges in 1936–1938. In June 1939, 1 year before Vavilov’s arrest, Prezent and Lysenko wrote an official complaint to the Chairman of the Council of People’s Commissars (Prime Minister), Viacheslav Molotov. They stated that Vavilov’s public utterances were anti-Soviet, and that Vavilov, together with other geneticists, “makes every effort to give the impression of science being persecuted in our country” (Vavilov 2003, p. 98). The complaint prompted Vavilov’s arrest in 1940. Even then, Prezent and Lysenko called for the prohibition of genetics, which had allegedly become a means to fight “against avant-garde science.”

After Vavilov’s arrest, Lysenko became head of the Institute of Genetics, and Iohann Eichfeld, who was a former student of Vavilov but took Lysenko’s side, became head of the VIR and dismissed virtually all of Vavilov’s followers. The number of researchers oppressed in the VIR alone was greater than the total number of biologists oppressed in Nazi Germany (Kolchinsky 2014). Two biologists were arrested and murdered, and 39 were dismissed for political and racial reasons in the Third Reich (Deichmann and Müller-Hill 1994). In the VIR, at least 10 leading researchers were arrested, and were shot or died in prison, 12 spent many years in prison or exile, and were then freed, and > 26 were dismissed after Vavilov’s arrest (Dragavtsev and Lebedev 1994). The list is far from complete.

These actions were interrupted in 1941, when the USSR joined the Second World War. After the war, the Central Committee of the Communist Party was flooded with letters from Soviet biologists from various specialties, including plant breeders, complaining of Lysenko’s totalitarian style, intolerance of criticism, and desire to take administrative measures against his opponents (Levina 1995). Geneticists were supported by their foreign colleagues, who accused Lysenko of pseudoscience. In 1946, Dobzhansky translated Lysenko’s book *Heredity and Its Variability* (Lysenko 1946), to allow his colleagues to evaluate Lysenko’s ideas themselves. A review published the same year characterized Lysenko’s work as “*a mixture of logical and illogical methods and procedures*” (Hudson and Richens 1946). In December 1945, leaders of the USSR Academy of Sciences protested against Lysenko’s election to its Presidium, pointing to his poor scientific reputation (Esakov *et al.* 1991).

**Figure 1:**
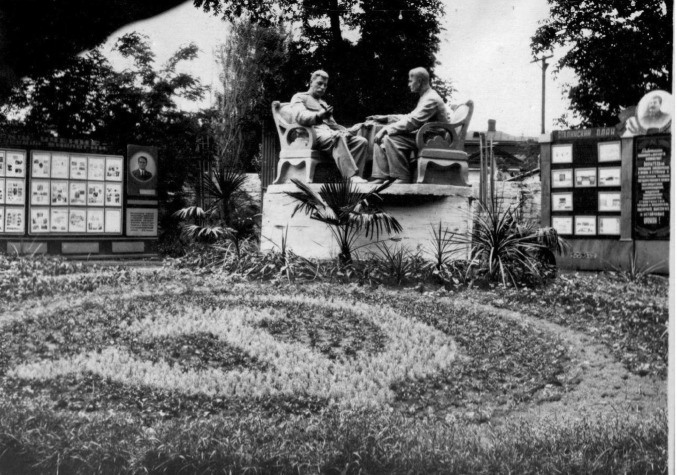
Sculpture of Stalin and Lysenko, which was built in Stavropol city in 1952 and demolished in 1961. Stalin held a branching wheat head in his hand. Courtesy of the Archive of Administration of Stavropol.

It is widely believed that formal geneticists (who won the sympathy of a number of powerful figures in USSR governmental circles and were supported by the world scientific community) almost succeeded in their opposition to Michurinists. That is not entirely true. Lysenko was guaranteed to win. The Cold War rendered everyone who showed solidarity with former allies in the anti-Hitler coalition suspicious in Stalin’s eyes. In addition, an explanation had to be provided for yet another postwar famine, which had taken the lives of ∼2 million people (Zima 1996), as well as names of people who could be condemned as obstacles to progress in agriculture. The situation again made Stalin favor Lysenko, who had promised new high-yield cultivars for the country, this time with the help of branching wheat bred from 200 g of seeds that Stalin had personally given him in late 1946 (Figure 1). Knowing that previous attempts to introduce branching wheat into agricultural production had failed, Lysenko promised to solve the problem in 2 or 3 years by retraining. Moreover, Lysenko’s belief that acquired traits were inherited was consistent with Stalin’s own Lamarckian views, while the postulate that the environment plays a crucial role in the transformation of organisms harmonized with Stalin’s concept of the transformation of nature and man. At that time, some non-Soviet biologists also advocated the inheritance of acquired traits, but Lysenko’s scientific standing, even among such biologists, was not sufficient to justify his rise.

Disagreements between Lysenko and most other biologists had by then increased, as Lysenko, who claimed to follow Darwin in words, departed from Darwinian theory by criticizing the concept of intraspecific competition and suggested direct adaptation, rather than selection, as the main factor in evolution. Leaders of several different biological disciplines criticized his views at conferences held at Moscow State University in November 1947 and February 1948. On November 4, 1947, Lysenko published an article denying intraspecific competition in the popular magazine *Literaturnaia gazeta* and, on November 29, Sergei Iudentsev, dean of the Biological Faculty of Moscow State University, the evolutionist Ivan Schmalhausen, the biogeographer Aleksandr Formozov, and the plant physiologist Dmitrii Sabinin responded criticizing Lysenko.

Seeking protection, Lysenko appealed to Stalin by letter (October 27, 1947). He reported his work with branching wheat and other projects, but the main part of the letter appealed for support against geneticists: “*Mendelism-Morganism*, *Weissmanist neo-Darwinism... are not developed in Western capitalist countries for the purposes of agriculture*, *but rather serve reactionary purposes of eugenics*, *racism*, *etc. There is no relationship between agricultural practices and the theory of bourgeois genetics*” [cited in Vavilov (2003)]. He insisted that administrative measures be taken to support Michurinist biology against research geneticists and in science education. Stalin agreed, responding (on October 31, 1947): “*As for theoretical concepts in biology*, *I think that Michurin’s concept is the sole concept that is scientific. Weissmanists and their supporters*, *who deny inheritance of acquired properties*, *do not deserve that we go on about them for long. The future belongs to Michurin*.” Stalin promised that the party would support Lysenko (Vavilov 2003) and personally reviewed Lysenko’s presentation for the forthcoming 1948 VASKhNIL session (Rossianov 1993).

## The August 1948 VASKhNIL Session and its Aftermath

**Figure 2:**
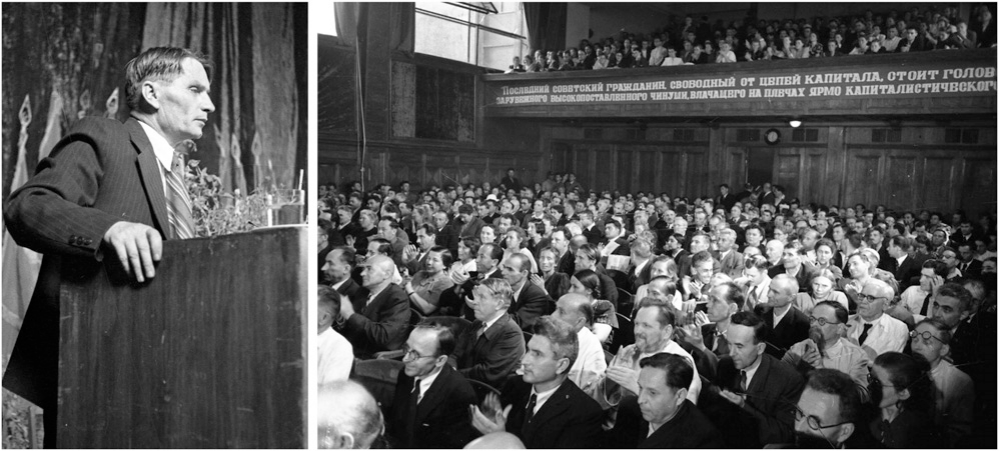
Trofim Lysenko and attendees of the August Session of the Vsesoiuznaia Akademiia Sel’skoKhoziaistvenenykh Nauk imeni Lenina. Courtesy of the Russian state archive of film and photo documents. The inscription on the banner reads “*The humblest Soviet citizen*, *being free from the fetters of capital*, *stands head and shoulders above any high-placed foreign bigwig whose neck wears the yoke of capitalist slavery*” (Stalin’s Report to the 18th Congress of the Communist Party, delivered March 10, 1939).

The historic 1948 session was carefully planned and prepared under Stalin’s instructions, and commenced at the Ministry of Agriculture on July 31, 1948 and continued until August 7. The session was attended by researchers, agronomists, livestock specialists, farm machinery operators, and economists, ∼700 people in total (Figure 2). Pavel Lobanov, a deputy minister of agriculture of the USSR, was chairman. Lysenko’s speech, “The Situation in Biological Science” (Stoletov *et al*. 1949), took up the whole first day. The next day was dominated by an excursion to Lysenko’s experimental station, Gorki Leninskie (Lenin Hills), where work with branching wheat was demonstrated. Lysenko promised Stalin to achieve the infeasible yield of 15,000 kg/hectares (ha) (Vavilov 1998); common wheat yields were 700–800 kg/ha at that time, reaching 2000 kg/ha in exceptionally favorable conditions (Sazonova 2007; Rastiannikov and Deriugina 2009).

In his speech, Lysenko rejected intraspecific competition, the nondirectional character of mutations, and “Mendelism-Morganism-Weissmanism.” He categorized statements claiming that a “substance of heredity” existed as reactionary and idealistic, while statements on heritability of acquired traits were materialistic, separating biology into two worlds with different political ideologies. The theoretical part of the speech was a mixture of animism and natural philosophy, with references to Jean Lamarck and citation of certain ideas of Ernst Haeckel (without mentioning his name), using concepts borrowed from writings about heredity and variation in the pregenetic era. Lysenko stated that Mendelism-Morganism was taught in departments of genetics, breeding, and Darwinism, which sidelined Michurinism. Many critical comments were directed against the evolutionist Ivan Schmalhausen, the geneticist Nikolai Dubinin, the plant breeder Petr Zhukovskii, and the protozoologist Iurii Polianskii, all of whom had openly criticized Lysenko in the previous 2 years. In conclusion, Lysenko encouraged the VASKhNIL to ensure that “*Michurin’s concepts are developed*… *as we are taught by the personal examples of attitude toward Michurin’s activity that are presented to us by our great teachers V.I. Lenin and I.V. Stalin*.” The concluding words were a mandatory ritual and were, as always, accompanied by a storm of applause.

*Pravda* published Lysenko’s speech on August 4. Until then, some of the participants were still under the illusion that the Communist party would not decide scientific matters. Geneticists did not recant, but defended their views. Without casting doubt on the achievements of Michurinist agrobiology, and instead expressing a readiness for dialogue with Michurinists, they advocated continued research in genetics and freedom of discussion of different scientific schools of thought. However, only 8 out of 56 speakers talked about these matters. Among these, geneticists Sos Alichanian and Anton Zhebrak said that a ban on genetics would damage agriculture. The botanist Petr Zhukovskii invited Lysenko to write a textbook on plant biology and promised to accept his method if it proved effective in practice, and the physiologist Boris Zavadovskii pointed out that millions of hectares had been sown with valuable wheat and rye cultivars developed by geneticists.

Vasilii Nemchinov, head of the Timiryasev Agricultural Academy (TSKhA, Timiryazev SelskoKhoziastvennaia Academia), an important center of research and education, rejected accusations that Michurinists had been driven out of the TSKhA and supported Zhebrak, the breeder Petr Konstantinov, and the evolutionist Aleksandr Paramonov, who worked at the TSKhA and had been attacked by Lysenkoists.

The geneticist Joseph Rapoport highlighted achievements in the field of chemical mutagenesis, which had offered opportunities for accelerating breeding. Rebutting the accusations that geneticists were anti-Darwinists, he reminded the participants that it was Lysenko’s Lamarckism that was incompatible with the theory of selection. He prophetically said that biology was at the threshold of understanding the nature of genes, which promised enormous practical outcomes.

Schmalhausen, whom Lysenko named a central “Weissmanist-Morganist” and an advocate for “bourgeois science,” denied such accusations, while describing the Darwinian interpretation of relationships between environmental factors, mutations, and adaptations, and emphasizing the crucial role of natural selection in evolution. He demonstrated Prezent’s and his associates ignorance of biology.

Lysenkoists aggressively responded to the advocacy of genetics, in language that was often abusive and inappropriate for a scientific discussion: “fly breeders,” “retrogrades,” “idealistic pseudobiology,” “an anti-people field of science,” and “a theory hostile to practice.”

Almost all the 48 other speakers, including 15 of the 35 newly appointed full VASKhNIL members, spoke enthusiastically about Lysenko’s address, his contributions to Michurinist biology, and its practical “advances.” Nearly every speaker delivered a rant against “Mendelists-Morganists-Weissmanists” and complained of being oppressed by geneticists, and by the teaching of Mendel’s, Weissman’s, and Morgan’s concepts in schools and higher education institutions, and about the obstacles to the implementation of Michurinist biology.

In contrast, the role of genetics in breeding was well understood by Iohann Eichfeld, the head of the VIR and a former student of Vavilov. However, being aware of the shift in the political situation, he claimed that he employed Lysenko’s methods (Stoletov *et al*. 1949). Pavel Luk’ianenko, another student of Vavilov who had obtained a rust-resistant high-yield wheat cultivar by genetic methods, similarly stated that his work had been based on Michurinist agrobiology (Stoletov *et al*. 1949). However, Luk’ianenko’s presentation revealed that leading breeders continued to rely on Vavilov’s scientific heritage, while pretending to work by Lysenko’s methods (Tauger 2017).

In Lysenko’s closing statement, he said that “*the Central Committee of the Communist Party has examined my report and approved it*” (Stoletov *et al*. 1949). Everyone was therefore aware that Lysenko’s concepts were now official doctrine and could not be criticized. Three of the eight scientists who had advocated genetics immediately made repentant statements. The others did the same later. Rapoport alone refused to admit “mistakes.” He was expelled from the Communist Party, a punishment considered more severe than dismissal from one’s employment. It took another 10 years before he was able to return to science. Lysenko’s address was issued as a pamphlet and 200,000 copies of the verbatim record of the session were printed by the end of August.

While in the 1930s there was still the illusion of discussions among scientists, the August 1948 session was a political and administrative event aimed at suppressing those who had not accepted the Michurinist biology approved by Stalin. As early as August 9, 1948, the Secretariat of the Central Committee of the Communist Party intervened in the main education institutions in agronomy and biology (Esakov 2000). The director of the TSKhA, Nemchinov, was replaced by Lysenko’s supporter Vsevolod Stoletov. The Biological Faculty of Moscow State University became the Biological and Soil Faculty, with Prezent appointed as its head in place of the dismissed Iudentsov. Schmalhausen was removed from his post as head of the Darwinism Department at Moscow State University and then relieved of all his duties at the USSR Academy of Sciences. Later on, orders to close departments and laboratories, and dismiss researchers, flooded in from ministries.

Higher education minister Sergei Kaftanov issued an order dated August 23, 1948, to dismiss everyone who had “*actively fought against Michurinists and Michurinist doctrine and failed to educate the Soviet youth in a spirit of progressive Michurinist biology*” (Kaftanoff 1948). Heads of higher education institutions were instructed to ensure “*a fundamental restructuring of educational and research activities to equip students and researchers with knowledge of the ground-breaking*, *progressive Michurinist doctrine and to vigorously root out the reactionary idealistic Weissmanist (Mendelist-Morganist) branch*.” Several textbooks on biology, including those on Darwinism by Paramonov and Schmalhausen, were withdrawn and destroyed. Mutant strains and various genetic preparations were also destroyed.

Academician Vladimir Strunnikov, head of the Commission for the History of the Development of Genetics in the Soviet Union at the USSR Academy of Sciences, wrote: “*In autumn 1948 alone*, *127 teachers*, *including 66 professors*, *were dismissed. The total number of those who had been dismissed*, *demoted*, *or removed from leadership positions after the session of the VASKhNIL of 1948 amounted to several thousands of people*” (Strunnikov 2004). Dozens of leading biologists, including the entire staff of the departments of genetics and Darwinism, were dismissed from Moscow State University. Michurinists headed by Prezent took over their offices. Prezent was additionally made head of the Department of Darwinism in Leningrad State University, which ensured that many of his opponents, including the university head Polianskii, were dismissed. Nikolai Turbin replaced the dismissed geneticist Mikhail Lobashev, and headed the reorganized Department of Genetics and Breeding in Leningrad State University. Zhebrak and Konstantinov were dismissed from the TSKhA, while Lysenko himself became head of the Department of Genetics and Breeding. Sergei Chetverikov, who was head of the Department of Genetics and Breeding in Gorki University, resigned his position, saying that he was unable to give up genetics: “*If I did this*, *nobody in the geneticists’ community would believe in that*” [cited in Philipchuk and Timkova (2017)]. More than 50 people were removed from their offices in Tomsk, a town in Siberia (Sizov 2004).

In Omsk, the mycologist Konstantin Murashinskii, who had refused to accept the results of the August session, was charged with adherence to fascism and preferred commiting suicide to being persecuted further. Aleksandr Promptov, a founder of Soviet ethology, and Dmitrii Sabinin, a renowned plant physiologist, also committed suicide. Others remained unemployed for a long time or tried to obtain a job in different fields. Yet no figures can in any way describe the tragedy that the majority of biologists experienced when forced to renounce their views and witness the ruin of their science. Moreover, as the war veteran Polianskii recollected when speaking in the Event Hall of Leningrad State University in spring 1987: “*You were not afraid for yourself*, *but feared that*, *failing to bear torture during interrogations*, *you would incriminate and ruin your friends and colleagues*.”

An extended meeting of the Presidium of the USSR Academy of Sciences was held on August 24–26, 1948. As the physiologist Leon Orbeli, who headed the Division of Biological Sciences, refused to repent, Aleksandr Oparin, famous for his theory of the origin of life, and a supporter of Lysenko, was elected to replace him. Nikolai Vavilov’s brother, the prominent physicist Sergei Vavilov, was president of the USSR Academy of Sciences and he “admitted” that Lysenko’s ideas were scientifically correct and promised that everything would be done to ensure that “*Michurinist biological science receives full development in biological institutes*, *journals*, *and publishing activity and that “reactionary-idealistic biology” is totally eliminated*” (Anonymous 1948). His personal position was evident from his diary note: “*Everything is so sad and shameful*” (Orel *et al.* 2012, p. 364). However, at the cost of this compromise, Sergei Vavilov was permitted to “clean away” anti-Michurinists internally and staff losses in the USSR Academy of Sciences were consequently not as great as in the higher education institutions.

The decisions of the August session had a ripple effect within the total scientific community, affecting areas distant from genetics, as well as biology in general. In 1950, sessions on physiology, cytology, and microbiology were held in the USSR Academies of Sciences and Medical Sciences to condemn the most important achievements in biology, and glorify the “experiments” of Olga Lepeshinskaia, who claimed to have observed cells emerging from unstructured vital substances, or Gevork Bosh’ian, who claimed to have “demonstrated” that viruses turn into bacterial cells, and bacteria into viruses and antibiotics. Cybernetics, mathematical logic, certain fields of physics and chemistry, sociology, economics, and even philology were jeopardized. Party ideologists chose a “Lysenko” as the sole holder of true knowledge for each discipline (Graham 1987, 17–18). Only the military significance of studies on nuclear energy prevented a planned session taking place to condemn quantum physics and relativity theory.

It is important to acknowledge that the leaders of Michurinist biology were also kept on a short leash by the authorities. Lysenko’s failures became obvious and were reported to Stalin. In 1951, its main ideologist Prezent was relieved of all his duties and expelled from the party with severe political accusations. Stalin soon allegedly made the pronouncement that: “*Lysenko should be forced to love criticism*” (Soyfer 1994). In 1952, with Stalin’s permission, *Botanicheskii zhurnal* (Botanic Journal) published Turbin’s article that criticized Lysenko’s views on species and speciation. These were ominous signs of a forthcoming fall from grace. However, Stalin died on March 5, 1953 and Khrushchev assumed power. Lysenko again promised to greatly increase agricultural yields and gained Khrushchev’s support. However, the general situation in the country, and in science, started to change, and the Party press called for freedom of discussion in science and elimination of dogmatism in biology (The editorial *Science and Life* in the Soviet Communistic Party journal *Kommunist*, (Anonymous 1954). A discussion about Lysenko’s views of species and speciation opened in several biological journals soon afterward, having being approved by the Central Committee of the Communist Party. The authorities were flooded with letters criticizing Lysenko from researchers in various disciplines and from agricultural workers, who knew how ineffective Lysenko’s techniques were. The field of genetics remained under a cloud, but two of its advocates, Armen Takhtadzhian and Kirill Zavadskii, headed the Biological and Soil Faculty in Leningrad State University from 1953 to 1955 (Kolchinsky 2013). Under them, biologists who had been expelled after the August 1948 VASKhNIL session returned to the university. Navashin started lecturing on genetics in 1955, while Lobashev, who succeeded him, published a genetics textbook in 1963, the first since the August session (Inge-Vechtomov 2015a; Kolchinsky and Shalimov 2017).

In the Soviet scientific community, there was a growing awareness that Lysenko’s monopoly was harmful not only to biology, but also to the image of the country and its defense. The discovery of the double helix made it clear, even to nonbiologists, that his ideas were absurd. In 1955, > 300 scientists from various specialties, including the future physics Nobel Prize winners, L. D. Landau, I. Ye. Tamm, and V. L. Ginzburg, and heads of nuclear and space programs, M. V. Keldysh, Y. B. Zel’dovich, and Y. B. Khariton, signed a letter to the Presidium of the Central Committee of the Communist Party demanding that Lysenko’s domination in biology be brought to an end. Three biologists, the cytologist V. Ya. Aleksandrov, D. V. Lebedev, a botanist and plant geneticist, and the geneticist Yu. M. Olenov, wrote the letter and collected signatures [see Zakharov *et al.* (2005)]. This action became possible owing to Khrushchev’s policies of de-Stalinization, accompanied by a decrease in repression and censorship. Researchers stood up for freedom in the development of science against dictation by the Party and government, and the authorities conceded. Lysenko was removed as VASKhNIL President, but continued as Khrushchev’s personal advisor on agriculture and it was still unsafe to criticize him. Nevertheless, in 1956–1957, genetic laboratories came to be organized in physical institutes of the USSR Academy of Sciences, while the Institute of Cytology and Genetics was organized in Novosibirsk.

In October 1964, the Central Committee removed Khrushchev from power and Lysenko lost government support. However, Michurinists continued to lead the VASKhNIL, and head departments and faculties in agricultural higher schools, and even several universities, and were still training new generations of supporters. Lobanov, who chaired the August session, was President of the VASKhNIL from 1965 to 1978.

## International Effect of the VASKhNIL Session

The August 1948 VASKhNIL session made Michurinist biology an international phenomenon. Political context affected the statements of scientists from countries belonging to different blocs during the Cold War (Krementsov 2000; Wolfe 2010; deJong-Lambert and Krementsov 2017). Eastern Bloc countries tried to follow Moscow’s policy toward science, and prosecutions of Mendelists started in some of them, including Bulgaria and, soon afterward, China (Schneider 2003; Edreva 2013). Almost all socialist countries required that Michurinist biology be taught, using Soviet textbooks or those written by local followers of Lysenko.

However, Michurinist biology was not supported in East Germany, where Georg Schneider, head of the Ernst Haeckel House, was almost the only researcher who tried to demonstrate the inheritance of acquired traits in axolotl (Hossfeld and Olsson 2002). In Hungary, criticism of Lysenkoism and the eradication of its consequences started as early as 1953 (Palló and Müller 2017). In Poland, Michurinist biology was initially introduced using administrative measures by the authorities, but articles advocating the doctrine disappeared from the mass media after 1956 (Köhler 2016). In China, genetics was officially rehabilitated in 1956, but geneticists, together with all other scientists, suffered persecution again soon afterward (Schneider 2003). In Czechoslovakia, most biologists assessed Lysenkoism negatively in the late 1950s (Simunek and Hosfeld 2013), but its enthusiastic proponent Victor Novak tried to reconcile Lysenkoism with the neo-Darwinian synthesis for the rest of his life (Hampl 2016). In Romania, Michurinist biology clung on until the mid-1960s (Oghina-Pavie *2017*).

In the English-speaking world, in contrast, the August 1948 session was received negatively (Paul 1983; Harman 2003; Wolfe 2010; Gordin 2012; Selya 2012), with terms including the “death of science in Russia,” “Soviet tyranny in science,” “Walpurgis Week in the Soviet Union,” “science in bondage” *etc*. (Cook 1949; Dobzhansky 1949; Huxley 1949; Zirkle 1949; Muller 1951). Hermann Muller had opposed Lysenko from the mid-1930s, but remained an adherent of communism. Having learned about the August session, he resigned from the USSR Academy of Sciences. A response from prominent British evolutionary geneticists was published in *The Listener magazine* on December 8, 1948. Three British geneticists, Cyril Darlington, Sydney Harland, and R.A. Fisher, criticized Lysenko’s views. J.B.S. Haldane’s communist convictions restrained him from making forceful comments about the August session for a time, but later he left the British Communist Party. The prominent British physiologist Henry Dale resigned from the USSR Academy of Sciences. A rare exception was James Fyfe, who wrote a book supporting Lysenko (Fyfe 1950).

In France and Germany, where Lamarckism was strong (Junker and Engels 1999; Grimoult 2000), the August 1948 session was criticized by biologists including Jean Rostand and Jacques Monod (Buican 1978). The leading West German geneticist Hans Nachtscheim (Nachtscheim 1952) called the August session unprecedented violence against biology. Italian geneticists used it to bring about organizational separation from Michurinists (Cassata 2017).

The reception of Michurinist biology in Japan was affected by anti-Americanism after the war and a lack of advanced genetics (Fujioka 2013; Iida 2015). “Left-wing scientists” viewed it as a symbol of cutting-edge science, while many were attracted by its claimed agricultural benefits. Moreover, what had actually taken place at the August session was not fully clear to the Japanese and Lysenko’s ideas were discussed seriously in the Japanese specialist literature until the mid-1950s. Even after that time, marginal works on the topic were published over a period of > 20 years.

Lysenkoism and its perception globally and in specific countries have been discussed in several recent symposia: The International Workshop on Lysenkoism (New York 2009), The Second International Workshop on Lysenkoism (Vienna 2012), Reconsidering the Lysenko Affairs (Tokyo 2012), From Lysenkoism to Evolutionary Biology (Prague 2016), and three presentations at The International Society for the History, Philosophy, and Social Studies of Biology Meeting (São Paulo 2017), as well as special issues of the *Journal for the History of Biology* (2012) and *Studies in the History of Biology* (2013, 2016). These showed that the past assessments of the August session’s outcomes depended primarily on researchers’ political preferences and the position that a country adopted during the Cold War, or on its advocacy of national *vs.* international science.

## Attempts to Reassess the VASKhNIL August 1948 Session in Modern Russia

Not long ago, it seemed that the rationale, activities, and consequences of the August session of the VASKhNIL had been thoroughly investigated and definitively assessed. However, since the early 21st century, several advocates of Lysenkoism are even now trying to rehabilitate it as a national variant of genetics for patriotic motives (*e.g.*, Kononkov 2014).

Publications have appeared reappraising Lysenko as a great scientist and reevaluating the 1948 session. This has coincided with officials’ attempts to deprive the VIR of its main building, and confiscate experimental stations and farms to build private mansions (Dragavtsev 2011). Although the main building has been defended, a campaign of discrediting Nikolai Vavilov was initiated, which intensified together with the spread of anti-Western rhetoric in the mass media and the statements of Russian politicians.

In 2014, Pyotr Kononkov, Honored Science Worker of the Russian Federation, laureate of the State Prize, student of Lysenko, and vegetable grower, published a book *Two Worlds*, *Two Ideologies*, whose title was a reference to Lysenko’s 1948 report. Kononkov portrayed Lysenko as a great scientist, who developed agriculture and saved his country from famine. He attributed to Lysenko, without substantiation, virtues that are popular in today’s Russia: an Orthodox worldview, patriotism, and loyalty to national values and Russian geopolitical interests. For geneticists, Kononkov offers labels including national traitors, pseudoscientists, and charlatans who are hostile to Russia’s global structures. The book was published with support from the State Program of the Ministry of Culture of the Russian Federation.

The most shocking statement from the scientific community came with the publication of the book *Unknown Lysenko*, also in 2014, written by a biostatistician from the Institute for General Genetics of the Russian Academy of Sciences, Lev Zhivotovsky. He claims that Lysenko was one of the founders of plant developmental biology and a pioneer of epigenetics, while equating the value of the scientific works of Lysenko’s followers with those of geneticists. Concerning the August session, he writes that: “*the geneticists themselves contributed to what happened*” (Zhivotovsky 2014). To validate these statements, Zhivotovsky ignores the facts and corrupts citations. For example, he ascribes to geneticists views bearing no relation to reality: “*A main methodological approach in the genetics of the 1920s and early 1930s was based on the assumption that all traits of a biological individual are predetermined by its genes*, *while the environment only passively provides for its feeding and other needs*” (Zhivotovsky 2014). It is relevant to recall that Hermann Muller discovered X-ray mutagenesis in 1926. Regarding the persecution of scientists supporting Lamarckism, Zhivotovsky writes: “*Attempts made by several researchers to prove the main postulates of Lamarckism did not gain support*, *but induced active opposition*, *which sometimes led to tragic outcome. Remember the story of P. Kammerer*, *who interpreted his findings as supporting heritability of acquired characteristics*, *was then accused of scientific falsifications by W. Bateson*, *and committed suicide*” (Zhivotovsky 2014). By failing to mention the details of Paul Kammerer’s work (Alphen and Arntzen 2016, 2017), a distorted image is given to young biologists who know little of this tragic case in the history of science.

The entomologist and Moscow University professor Anatolii Shatalkin regards the August 1948 session as a “*pogrom* [that was] *destructive for science*” but puts the blame, not on Stalin and Lysenko, but on ideologists from the Central Committee of the Communist Party, Anglo-American scientists, and the US Department of State for playing off Michurinists against the geneticists (Shatalkin 2015). This conspiratorial view corresponds to the Stalinist image of a Russia surrounded by enemies, which is being revived today.

The Russian scientific community reacted with a wave of critical publications written by geneticists and historians of science [Golubovsky (2015), Inge-Vechtomov (2015b), Zakharov-Gezekhus (2015), and other authors]. Digests of this criticism have also been published (Graham 2016; Kolchinsky 2017), while the mass media are again involved in this controversy. Pro-Lysenko articles are, even now, being published by conservative and patriotic newspapers, such as *Literaturnaia gazeta*, *Kulturae*, and *New Novyi Peterburg*, although critical responses do appear in the *Rossiiskaia gazeta*, *Novaia gazeta*, and *Troitskii Variant*. Television does not stand aside either.

The attempts to reassess the VASKhNIL August session are influenced by various factors. In particular, the promotion of Lysenkoism and other pesudoscientific ideas in the mass media (and sometimes lobbied for by state structures) are occurring in a time of a decline in the status of science, actions damaging to institutional reputations, and falling educational standards. The August 1948 VASKhNIL session is fondly remembered by those who feel nostalgic about Stalin’s “iron-fisted” rule, while admirers of the Lysenkoists crave a return to the old Soviet days. Although most monuments to Stalin were destroyed under Khrushchev in the 1960s, his growing popularity is reflected by the fact that > 100 new statues have been installed since the 2000s on the initiative of local divisions of the Russian Federation Communist Party or by individuals. Space to erect a statue has sometimes been provided by local authorities. Rehabilitation of Lysenko is part of the same process. The authors of pro-Lysenko writings have different motives, but they all studied from textbooks written by the Lysenkoists, and began their careers in institutes controlled by Lysenkoists’ students and followers. It is fully understood by most Russian scientists that Michurinist biology was long-ago consigned to history (Kolchinsky *et al.* 2017), but science is again out of favor among Russia’s rulers, giving the opportunity for Lysenko’s reincarnation.

## Conclusions

The August session of the VASKhNIL triggered a series of propaganda campaigns on both sides of the Iron Curtain to demonstrate the incompatibility of the East and the West, not only in politics and ideology, but also in science. Stalin sanctioned the campaign to consolidate the socialist bloc, to mobilize its politics, economics, ideology, and culture, and to explain to the population why agriculture was in a pitiful state. He rejected the idea of international science, and made Soviet scientists align themselves with fictitious or real indigenous versions. Geopolitically, measures taken after the session were designed to strengthen the dividing line in politics and culture by demonizing those who stood to integrate global science. Lysenko, as the organizer of the August 1948 session, is often called the diabolical genius of Soviet biology. However, he was only Stalin’s minion. If Stalin had not directed it, the session would have merely been one of the scandalous campaigns of that time, affecting only the VASKhNIL. However; Stalin’s involvement gave it global significance.

The main consequence of the August session was the underdevelopment of Russian biology. While the DNA double helix was discovered and molecular biology emerged abroad, research standards and methods from, at best, half a century earlier were forced on Russian biologists. The impossibility of communicating with foreign colleagues and lack of training made Russia lag even further behind in the most promising fields of genetics research. The consequences still persist because several generations of biologists grew up during the dominance of Michurinist biology.

Viewed more broadly, the August session of the VASKhNIL was part of a centuries-long debate in Russia about national *vs.* global culture. In the 1940s, this debate involved not only biology, but also science more generally. The departure from international research in genetics was especially devastating, affecting not only education and research, but also the provision of food. A detailed study of the background and consequences of the August session as an example of how dramatically the authorities may affect science makes it possible to understand the mechanisms that limit the free development, and discussion, of scientific ideas. Such an understanding is still important.

Lysenkoism provides the most dramatic, but not the only, example of how the authorities and ideology interfered with science in the Soviet Union with catastrophic consequences for biology, and the fates of researchers and society. Lysenkoism was a phenomenon distinct to the Soviet Union, though it then spread to other states in the Communist bloc at the time. However, there are still social forces and governments that try to regulate certain areas of science, and science teaching, in accordance with their ideologies. Thus, the dangers of state interference in science remains real, hence the history of those events remains relevant.

## References

[bib2] Alphen J. J. M. v. Arntzen J. W., 2016 Paul Kammerer and the inheritance of acquired characteristics. Contrib. Zool. 85: 457–470.

[bib3] Alphen J. J. M. v. Arntzen J. W., 2017 The case of the midwife toad revisited. Contrib. Zool. 86: 261–272.

[bib90] Anonymous, 1932 Minutes of the meeting of activists of the Society of biologists of February 11, 1932. Archive of the Russian Academy of Sciences St.Petersburg Branch, F. 240. Op. 1: D. 3. Ll. 1–5; D. 22. L. 12.

[bib5] Anonymous, 1948 An extended meeting of the Presidium of the USSR Academy of Sciences on 24–August 26, 1948 (in Russian). Available at: http://textarchive.ru/c-1860864-pall.html/. Accessed: July 03, 2018.

[bib6] Archive of the Russian Academy of Sciences St.Petersburg Branch F. 240. Op. 1: D. 3. Ll. 1–5; D. 22. L. 12.

[bib91] Anonymous, 1954 Science and Life (Editorial). Kommunist. 5: 3–13 (in Russian).

[bib7] Bateson W., 1925 Science in Russia. Nature 116: 681–683. 10.1038/116681a0

[bib8] Bengtsson B. O. Tunlid A., 2010 The 1948 International Congress of genetics in Sweden: people and politics. Genetics 185: 709–715. 10.1534/genetics.110.119305 20660651 PMC2907196

[bib9] Buican D., 1978 L’eternel retour de Lyssenko. Copernic, Paris.

[bib10] Cassata Fr., 2017 Lysenko in Bellagio: the Lysenko controversy and the struggle for authority over Italian genetics (1948–1956), pp. 37–72 in The Lysenko Controversy as a Global Phenomenon. Genetics and Agriculture in the Soviet Union and Beyond, Vol. 2, edited by de Jong-Lambert W. Krementsov N. L. Palgrave Macmillan, Basingstoke, UK 10.1007/978-3-319-39179-3_2

[bib11] Cook R., 1949 Walpurgis week in Soviet Union. Sci. Mon. 68: 367–372. 18150144

[bib12] Davies R. W. Wheatcroft S. G., 2004 The Years of Hunger: Soviet Agriculture, pp. 1931–1933. Palgrave Macmillan, Basingstoke, UK.

[bib13] Deichmann U. Müller-Hill B., 1994 Biological research at universities and Kaiser Wilhelm Institutes in Nazi Germany, pp. 160–183 in *Science*, *Technology*, *and National Socialism*, edited by Renneberg M. Walker M. Cambridge University Press, Cambridge, UK.

[bib14] deJong-Lambert W. Krementsov N. L. (Editors), 2017 The Lysenko Controversy as a Global Phenomenon Genetics and Agriculture in the Soviet Union and Beyond, Vol. 1–2 Palgrave Macmillan, Basingstoke, UK.

[bib15] Dobzhansky T., 1949 The suppression of a science. Bull. At. Sci. 5: 144–146. 10.1080/00963402.1949.11457065

[bib16] Dragavtsev V. A. Lebedev D. V. (Editors), 1994 Colleagues of Nikolai Ivanovich Vavilov. Researchers of Plant Gene Pool, VIR, St-Petersburg, Russia (in Russian).

[bib89] Dragavtsev V.A., 2011 Nikolai Ivanovich Vavilov is one of the 100 great people of the planet Earth. *Gatchinskaia Pravda*, Oct 25, p. 6 (in Russian).

[bib17] Edreva A., 2013 The destroying of an eminent geneticist: Dontcho Kostoff and the biological conference in Bulgaria, 1949. Stud. Hist. Biol. 5: 54–62.

[bib18] Efroimson, V. P., 1989 About Lysenko and Lysenkovshchine. Voprosy istorii estestvoznaniia i tekhniki 1: 79–93; 2:132–147; 3: 96–109; 4: 100–111 (in Russian).

[bib19] Elina O., 2017 Lysenko’s predecessors: the Demchinskys and the bed cultivation of cereal crops, pp. 37–66 in The Lysenko Controversy as a Global Phenomenon. Genetics and Agriculture in the Soviet Union and Beyond, Vol. 1, edited by de Jong-Lambert W. Krementsov N. L. Palgrave Macmillan, Basingstoke, UK 10.1007/978-3-319-39176-2_2

[bib20] Esakov V. D. (Editor), 2000 Academy of Sciences in the Decisions of the Politburo of the Central Committee of the RCP(b)- SUCP(b) 1922–1952. ROSSPEN, Moscow.

[bib21] Esakov V. D. Levina E. S. (Editors), 1987 Nikolai Ivanovich Vavilov. From the Epistolary Heritage of 1929–1940, Nauka, Moscow.

[bib22] Esakov V. D. Ivanova S. Levina E. S., 1991 From the history of the fight against Lysenkoism. Izvestiia TsK KPSS 4: 125–141.

[bib23] Fujioka T., 2013 The Japanese Lysenkoism and the historical backgrounds. Stud. Hist. Biol. 5: 7–15. http://shb.nw.ru/wp-content/uploads/2018/07/SHB-1-2013.pdf

[bib24] Fyfe J. L., 1950 Lysenko is Right. Lawrence & Wishart, London.

[bib25] Golubovsky M. D., 2015 The Ghost of Lysenko and his modern incarnation. Stud. Hist. Biol. 7: 114–130 (in Russian). http://shb.nw.ru/wp-content/uploads/2018/07/SHB-2015-2.pdf

[bib26] Goncharov N. P. Savel’ev N. I., 2016 Ivan V. Michurin: on the 160^th^ anniversary of the birth of the Russian Burbank. Russian Journal of Genetics: Applied Research 6: 105–127. 10.1134/S2079059716010068

[bib27] Gordin M. D., 2012 How Lysenkoism became pseudoscience: Dobzhansky to Velikovsky. J. Hist. Biol. 45: 443–468. 10.1007/s10739-011-9287-3 21698424

[bib28] Graham L., 1987 Science and Philosophy in the Soviet Union. Columbia Univ. Press, New York.

[bib29] Graham L., 2016 Lysenko’s Ghost. Epigenetics and Russia. Harvard Univ. Press, Cambridge, MA. 10.4159/9780674969025

[bib30] Grimoult C., 2000 Histore de l’Évolutionnisme contemporain en France: 1945–1995, Librairie Droz S. A., Genève, Switzerland 10.3917/droz.grimo.2000.02

[bib31] Hampl P., 2016 The evolution of theoretical views of Vladimír Novák: from Lysenkoism to epigenetics. Stud. Hist. Biol. 8: 11–24. http://shb.nw.ru/wp-content/uploads/2018/07/%D0%A2%D0%BE%D0%BC-8-%E2%84%963.pdf

[bib32] Harman O. S., 2003 C.D. Darlington and the British and American reaction to Lysenko and the Soviet conception of science. J. Hist. Biol. 36: 309–352. 10.1023/A:1024483131660 12945539

[bib33] Hossfeld U. Olsson L., 2002 From the modern synthesis to Lysenkoism, and back? Science 297: 55–56. 10.1126/science.1068355 12098687

[bib34] Hudson P. S. Richens R. H., 1946 The New Genetics in Soviet Union. School of Agriculture, Cambridge, UK.

[bib35] Huxley J., 1949 Soviet Genetics and World Science: Lysenko and the Meaning of Heredity. Chatto and Windus, London.

[bib36] Iida K., 2015 A controversial idea as a cultural resource: the Lysenko controversy and discussions of genetics as a ‘democratic’ science in postwar Japan. Soc. Stud. Sci. 45: 546–569. 10.1177/0306312715596460 26502659

[bib37] Inge-Vechtomov S. G., 2015 a *Genetics in retrospect*. N-L, St.-Petersburg (in Russian).

[bib38] Inge-Vechtomov S. G., 2015 b The book, after which I want to wash my hands. Stud. Hist. Biol. 7 (2): 109–112 (in Russian). http://shb.nw.ru/wp-content/uploads/2018/07/SHB-2015-2.pdf

[bib39] Joos L., 2017 State officials and would-be scientists: how the Ukrainian Ministry of agriculture discovered for Lysenko that he had made a scientific discovery, pp. 67–96 in The Lysenko Controversy as a Global Phenomenon. Genetics and Agriculture in the Soviet Union and Beyond, Vol. 1, edited by DeJong-Lambert W. Krementsov N. L. Palgrave Macmillan, Basingstoke, UK 10.1007/978-3-319-39176-2_3

[bib40] Junker T. Engels E.-M. (Editors), 1999 Die Entstehung der Synthetischen Theorie. Beiträge zur Geschichte der Evolutionsbiologie in Deutschland 1930–1950, Verlag für Wissenswchaft und Bildung, Berlin.

[bib92] Kaftanoff S., 1948 Order of the USSR Ministry of Higher Education no. 1208, August 23, 1948 On the state of teaching of biological disciplines in the universities and measures to strengthen the biological faculties by qualified staff of biologists-Michurinists (in Russian). Available at: http://www.libussr.ru/doc_ussr/ussr_4710.htm. Accessed July 03, 2018.

[bib41] Köhler P., 2016 Lysenkoist Propaganda in Trybuna Ludu. Stud. Hist. Biol. 8: 25–42. http://shb.nw.ru/wp-content/uploads/2018/07/%D0%A2%D0%BE%D0%BC-8-%E2%84%963.pdf

[bib42] Kolchinsky E. I., 2013 Kirill Mikhailovich Zavadskii: 1910–1977. Nestor–Istoriia, St. Petersburg, Russia (in Russian).

[bib43] Kolchinsky E. I., 2014 Nikolai Vavilov in the years of Stalin’s ‘revolution from above’ (1929–1932). Centaurus 56: 330–358. 10.1111/1600-0498.12059

[bib44] Kolchinsky E. I., 2017 Current attempts at exonerating ‘Lysenkoism’ and their causes, pp. 207–236 in The Lysenko Controversy as a Global Phenomenon. Genetics and Agriculture in the Soviet Union and Beyond, Vol. 2, edited by DeJong-Lambert W. Krementsov N. L. Palgrave Macmillan, Basingstoke, UK 10.1007/978-3-319-39179-3_8

[bib45] Kolchinsky E. I. Shalimov S. V., 2017 The thaw and genetics: the history of publishing of first Soviet textbooks on genetics. Rossiiskaiia istoria 4: 75–83 (in Russian).

[bib46] Kolchinsky E. I. Kutschera U. Hossfeld U. Levit G. S., 2017 Russia’s new Lysenkoism. Curr. Biol. 27: R1042–R1047. 10.1016/j.cub.2017.07.045 29017033

[bib47] Kondrashin B., 2008 *Famine of 1932–1933. The Tragedy of the Russian Village*. ROSSPEN, Moscow (in Russian).

[bib48] Kononkov P. F., 2014 *Two Worlds*, *Two Ideologies. On the Situation in Biological Sciences in Russia in the Soviet and Post-Soviet Period*, Luch, Moscow. (in Russian).

[bib49] Krementsov N. L., 1997 Stalinist Science, Princeton University Press, Princeton, NJ.

[bib50] Krementsov N. L., 2000 Lysenkoism in Europe: export — import of the Soviet model, pp. 179–202 in *Academia in Upheaval. Origins*, *Transfer and Transformations of the Communist Academic Regime in Russia and the East Central Europe*, edited by David-Fox M. Péteri G. Bergin and Garvey, London.

[bib51] Levina E. S., 1995 *Vavilov*, *Lysenko*, *Timofeev-Ressovskii. Biology in the USSR: History and Historiography. Airo-XX*, *Moskow.Lysenko*, *T. D*., *and I.I. Prezent 1935 Selection and Theory of Stage Development*. Selkhozgiz, Moscow (in Russian).

[bib52] Lysenko, T. D., 1936 Speech at the Meeting of the foremost grain yield, tractor and thresher drivers with the leaders of the party and the government. *Pravda*, January 2, 1936, no. 2 (6608): 3. (in Russian). Available at: https://pravda36.files.wordpress.com/2018/08/pravda-1936-2.pdf. Accessed: April 2, 2019.

[bib53] Lysenko T. D., 1946 Heredity and Its Variability. King’s Crown Press, New York.

[bib54] Lysenko T. D. Prezent I. I., 1935 Selection and Theory of Stage Development. Selkhozgiz, Moscow (in Russian).

[bib55] Medvedev Z. A., 1969 The Rise and Fall of T.D. Lysenko. Columbia Univ. Press, New York.

[bib56] Muller H. J., 1951 Science in bondage. Science 113: 25–29. 10.1126/science.113.2924.25 14798372

[bib57] Nachtcheim H., 1952 The Soviet violation of biology. J. Hered. 43: 19–21. 10.1093/oxfordjournals.jhered.a106249

[bib58] Nurinov, A., 1937 On a liberal note. The meeting of the asset of the Academy of Agricultural Sciences. *Sotsialisticheskoe zemledelie*, April 3, no. 76 (2464): 2 (in Russian).

[bib59] Oghina-Pavie Cr., 2017 The National pattern of Lysenkoism in Romania, pp. 73–102 in The Lysenko Controversy as a Global Phenomenon. Genetics and Agriculture in the Soviet Union and Beyond, Vol. 2, edited by deJong-Lambert W. Krementsov N. L. Palgrave Macmillan, Basingstoke, UK 10.1007/978-3-319-39179-3_3

[bib60] Orel V. M. Vavilova V. V. Krivonosov Yu. I., 2012 *Sergei Ivanovich Vavilov. Diaries. 1909–1951*, *book 2*. Nauka, Moscow (in Russian).

[bib61] Palló G. P. Müller M., 2017 Opportunism and enforcement: Hungarian reception of Michurinist biology in the cold war period, pp. 3–36 in The Lysenko Controversy as a Global Phenomenon, Vol. 2, edited by DeJong-Lambert W. Krementsov N. L. Palgrave Macmillan, Basingstoke, UK 10.1007/978-3-319-39179-3_1

[bib62] Paul D. A., 1983 War on two fronts: J. B. S. Haldane and the response to Lysenkoism in Britain. J. Hist. Biol. 16: 1–37. 10.1007/BF00186674 11611245

[bib63] Philipchuk I. V. Timkova S. V., 2017 The August session of the VASKHNIL (1948) and the massacre of genetics in the Gorky region pp. 179–184 in Issues of Archival and Source Studies in Higher Education. The Arzamas Branch of NNGU, Arzamas, Russia (in Russian).

[bib64] Pringle P., 2009 The Murder of Nikolai Vavilov. The Story of Stalin’s Persecution of One of Great Scientists of the Twentieth Century. Simon and Schuster, New York.

[bib65] Rastiannikov V. G. Deriugina I. V., 2009 Grain Crop Productivity in Russia (1795 – 2007). Oriental Institute, Russian Academy of Sciences, Moscow (in Russian).

[bib66] Reznik E. S., 2017 *This Short Life. Nikolai Vavilov and his time*. Zakharov, Moscow (in Russian).

[bib67] Rokitianskii Ia. G. Vavilov Yu. N. Goncharov V. F., 1999 The Court Executioner. Nikolai Vavilov in the dungeons of the NKVD: a Biographical sketch. Documents. Academia, Moscow (in Russian).

[bib68] Roll-Hansen N., 2005 *The Lysenko Effect. The Politics of Science*. Humanity Books, Amherst, NY.

[bib69] Rossianov K. O., 1993 Editing nature: Joseph Stalin and the “new” Soviet biology. Isis 84: 728–745. 10.1086/356638 8307727

[bib70] Sazonova L. V., 2007 Grain crop in Russia in 20th century: widening of the distribution and increasing of yields through the development of national breeding. Bulletin of Applied Botany, Genetics and Plant Breeding at N. I. Vavilov All-Russian Institute of Plant Genetic Resources 164: 379–392 (in Russian).

[bib71] Schneider L., 2003 Biology and Revolution in Twentieth Century China. Rowman & Littlefeld, Landham, MD.

[bib72] Selya R., 2012 Defending scientific freedom and democracy: the genetics society of America’s response to Lysenko. J. Hist. Biol. 45: 415–442. 10.1007/s10739-011-9288-2 21681530

[bib73] Shatalkin A. I., 2015 The Relational Concepts of Heredity and the Struggle Around Them in the 20^th^ Century. Scientific Press KMK, Moscow (in Russian).

[bib74] Simunek M. Hosfeld U., 2013 Trofim D. Lysenko in Prague 1960: a historical note. Stud. Hist. Biol. 2: 84–88. http://shb.nw.ru/wp-content/uploads/2018/07/SHB-2013-2.pdf

[bib75] Sizov, S. G., 2004 Regional bodies of the CPSU(b) – CPSU and the intelligentsia of the Western Siberia in 1946–1964. Ph.D. Thesis, Omsk State Technical University, Omsk, Russia (in Russian).

[bib76] Soyfer V. N., 1994 T.D. Lysenko and the Tragedy of Soviet Science. Rutgers Univ. Press, New Brunswick, NJ.

[bib94] Sstoletov, V. N., A. M. Sirotkin, and A. M. Ob’edkov (Editors), 1949 The situation in biological science: Proceedings of the Lenin Academy of Agricultural Sciences of the USSR. Session July 31 - August 7, 1948: Verbatim report, 1949. Foreign languages publ. house, Moscow.

[bib77] Strunnikov V. A., 2004 Silk Road. Nauka, Moscow.

[bib78] Tauger M., 2001 Natural Disasters and Human Action in the Soviet Famine of 1931–1933. University of Pittsburgh, Pittsburgh 10.5195/CBP.2001.89

[bib79] Tauger M. B., 2017 Pavel Pantelimonovich Luk’ianenko and the origins of the Soviet green revolution, pp. 97–127 in The Lysenko Controversy as a Global Phenomenon. Genetics and Agriculture in the Soviet Union and Beyond, Vol. 2, edited by deJong-Lambert W. Krementsov N. L. Palgrave Macmillan, Basingstoke, UK 10.1007/978-3-319-39176-2_4

[bib93] Vavilov N. I., 1930 American Philosophical Society Library B:D65 Th. Dobzhansky Papers. Vavilov, no. 1.

[bib81] Vavilov Y. N., 1998 The exchange of letters between T. D. Lysenko and I. V. Stalin in October 1947. Voprosy istorii estestvoznaniia I tekhniki 2: 153–165.

[bib82] Vavilov Y. I., 2003 Long Search: Book about Brothers Nikolai and Sergei Vavilovs. FIAN, Moscow.

[bib83] Wolfe A., 2010 What does It mean to go public? The American response to Lysenkoism. Hist. Stud. Nat. Sci. 40: 48–78. 10.1525/hsns.2010.40.1.48 20514743

[bib84] Zakharov I. K. Shumnyi V. K. Zhimulev I. F. Dubinina L. G., 2005 For the 50^th^ anniversary of “letter of three hundred”. Bulletin. VOGIS Vestnik 9: 12–33 (in Russian).

[bib85] Zakharov-Gezekhus I. A., 2015 The science of heredity in a distorting mirror of pseudoscience. V zashchitu nauki 16: 85–90 (in Russian).

[bib86] Zhivotovsky L. A., 2014 Unknown Lysenko. KMK Scientific Press, Moscow (in Russian).

[bib87] Zima V. F., 1996 Famine in the USSR 1946–1947. Origins and Consequences. IRAN, Moscow (in Russian).

[bib88] Zirkle C. (Editor), 1949 Death of Science in Russia. Univ. of Pennsylvania Press, Philadelphia 10.9783/9781512809060

